# Reclassification of variants of uncertain significance by race, ethnicity, and ancestry for patients at risk for breast cancer

**DOI:** 10.3389/fonc.2025.1455509

**Published:** 2025-02-18

**Authors:** Versha Pleasant, Jordyn Boggan, Blair Richards, Kara J. Milliron, Kristen S. Purrington, Michael Simon, Sofia Merajver

**Affiliations:** ^1^ Department of Obstetrics and Gynecology, University of Michigan, Ann Arbor, MI, United States; ^2^ Michigan Institute for Clinical and Health Research, University of Michigan, Ann Arbor, MI, United States; ^3^ Department of Internal Medicine, University of Michigan, Ann Arbor, MI, United States; ^4^ Department of Oncology, Karmanos Cancer Institute, Wayne State University, Detroit, MI, United States

**Keywords:** breast cancer, cancer genetics, variants of uncertain significance, race, ethnicity, ancestry, genetic testing

## Abstract

**Introduction:**

Although most variants of uncertain significance (VUS) in breast cancer susceptibility genes are eventually downgraded to benign or likely benign in individuals of European ancestry, it is unclear if this also applies to non-European populations. This study examines the time to and type of VUS reclassification among a diverse cohort at risk for breast cancer.

**Methods:**

A multicenter retrospective analysis examined people assigned female at birth (AFAB) who underwent genetic testing from 2013 to 2021 with VUS in *ATM*, *BARD1*, *BRCA1/2*, *CDH1*, *CHEK2*, *NF1*, *PALB2*, *PTEN*, *RAD51C/D*, *STK11*, and/or *TP53*. Demographic data were collected [including race, ethnicity, and ancestry (REA)], as well as time to and type of reclassification. Frequency data and univariable and multivariable analyses were performed (*p* < 0.05 was considered statistically significant).

**Results:**

There were 932 participants who had a total of 1,032 VUS (905 unique variants), with 20% who underwent reclassification of their results. The proportion of reclassified VUS among the largest represented REA groups was 19%, 23%, and 27% for White, Black or African American, and Asian people, respectively. REA was not associated with VUS reclassification (*p* = 0.25). The mean time to VUS reclassification was 2.8 years and was not significantly associated with REA (*p* = 0.16). Most VUS were downgraded to benign/likely benign (*n* = 187, 92%).

**Discussion:**

Our findings demonstrate that REA is not significantly associated with VUS reclassification or time to reclassification, with the majority of VUS being downgraded across REA. This study allows for improved and more equitable genetic counseling. It may also provide more reassurance to those groups that may have a higher likelihood of VUS results.

## Introduction

Germline genetic testing represents a key pillar of precision medicine. Regarding breast cancer risk, genetic testing could allow for the identification of high-risk individuals. Intensive surveillance with yearly mammogram and supplemental breast magnetic resonance imaging (MRI), along with options for chemoprevention and surgical risk reduction, may be available to those with pathogenic/likely pathogenic variants in breast cancer susceptibility genes. However, for patients with variants of uncertain significance (VUS), these clinical options are generally not applicable. VUS represent equivocal, non-actionable results that are monitored over time for possible reclassification as new data become available. Such results could cause distress among patients and confusion regarding interpretation (1–3). VUS also require additional follow-up from the ordering provider should a reclassification occur.

Current data suggest that those of non-European ancestry may have a higher prevalence of VUS compared to those of European ancestry (4–13). These disparities could be due to the lack of inclusion of non-European populations in familial studies, compounded with the lack of diversity in genome-wide association studies (GWAS) (14–17). There is also an overall lower uptake of genetic counseling and testing among certain populations, such as Black and African American communities (18, 19). This may in part be due to medical mistrust (20–22) but could also be a consequence of lower rates of physician referral (23–25). In addition, it is theoretically predicted that older human populations, such as those from sub-Saharan Africa, will experience more variability in single nucleotides (26).

The investigation of individuals of European ancestry showed that most VUS are expected to be eventually reclassified to benign/likely benign (27, 28). However, such prediction cannot necessarily be extrapolated to those of non-European ancestry who are largely underrepresented in genomic research. There is a dearth of data evaluating VUS reclassifications over time, particularly among diverse populations. One multisite study examining VUS rates among people susceptible to breast, ovarian, and colorectal cancer found that 8.1% of the total VUS results were reclassified. Regarding the type of reclassification, 88.7% were downgraded to benign/likely benign and 11.3% were upgraded to pathogenic/likely pathogenic clinically actionable variants. VUS reclassification rates were higher for Black people in comparison to the prevalence of this racial group of the entire sample (19.0% vs. 13.6%), lower for Asian people (3.5% vs. 6.3%), and proportional for White and Hispanic people (29). A cohort study evaluating VUS reclassifications among a variety of pathologic domains (including hereditary cancer) demonstrated that VUS were more commonly identified in people of non-European ancestry (30); however, reclassification frequency and type were not stratified by race, ethnicity, or ancestry (REA). There are no studies to date specifically and exclusively focusing on VUS reclassification by REA among individuals assigned female at birth (AFAB) who are at risk for breast cancer. The objectives of the current study were to assess the frequency of VUS reclassification and the length of time to reclassification by REA in a diverse cohort of AFAB people at risk for breast cancer and to determine the distribution of reclassification results.

## Materials and methods

This multicenter retrospective analysis included AFAB people who had VUS results following genetic testing between the years of January 2013 to December 2021 at the University of Michigan and Karmanos Cancer Institute (KCI) in Ann Arbor and Detroit, Michigan, respectively. The Breast and Ovarian Cancer Risk Evaluation Clinic is housed at the main campus of the University of Michigan in Ann Arbor, Michigan. First established in 1994, this clinic provides genetic testing and comprehensive lifetime risk assessment, education on anticancer lifestyle interventions, and clinical recommendations for patients at increased risk of cancer or with hereditary cancer syndromes. This clinic serves all 83 counties throughout the state of Michigan, for which reach was increased after COVID-19 due to a switch to virtual visits. Located in Detroit, the KCI is designated as the Comprehensive Cancer Center by the National Cancer Institute. It is affiliated with a network of 14 community sites across Michigan, which together make up the McLaren Medical Network. Individuals were evaluated by the Cancer Genetic Counseling Service (CGCS) in Detroit and in seven other McLaren community sites. Individuals from both sites were offered genetic testing if they met criteria based on the National Comprehensive Cancer Network (NCCN) guidelines.

Only AFAB people ≥18 years old were included in the analysis. Data collection included medical record abstraction for participants at the University of Michigan and abstraction of de-identified genetics registry data for KCI participants. Individuals who had VUS in any one of the following genes at the time of their initial cancer genetic testing were included in the analysis: *ATM*, *BARD1*, *BRCA1*, *BRCA2*, *CDH1*, *CHEK2*, *NF1*, *PALB2*, *PTEN*, *RAD51C*, *RAD51D*, *STK11*, and *TP53*. These specific genes were selected as they are known to increase the lifetime risk of breast cancer and were determined to be clinically actionable as per NCCN guidelines (31) during the data collection period for this project. The year of testing, testing laboratory, and number of genes tested in each panel were collected. Of note, the names of specific gene panels were not included in this study given that broader panels could have been utilized based on the participant’s personal and family cancer history (beyond just breast cancer, for instance). Furthermore, it is recognized that specific gene panel names and number of genes tested may change over time.

Demographic information was collected on self-reported REA, age at the time of testing, and personal or family history of breast and other cancers. This study used the National Institutes of Health (NIH) racial and ethnic categories classification, which includes American Indian or Alaska Native (a person having their origins from original peoples of North or South America and maintains tribal affiliation), Asian (a person having origins from peoples from the Far East, Southeast Asia, or the Indian subcontinent, which includes Cambodia, China, India, Japan, Korea, Malaysia, Pakistan, the Philippines, Thailand, and Vietnam), Black or African American (AA) (people of the African diaspora), Hispanic or Latino (a person having origins from Cuba, Mexico, Puerto Rico, South or Central American, or another Spanish culture or origin), Native Hawaiian or Other Pacific Islander (a person having origins from Hawaii, Guam, Samoa, or other Pacific Islands), or White (a person having origins from Europe, the Middle East, or North Africa) (32, 33). The use of White or European ancestry as a comparison group was chosen based on prior reclassification data being predominantly extrapolated from these particular REA groups.

More specific data on ancestry and family origins were ascertained for some in the study cohort but were not consistent across time and institutions (for instance, regarding the specific country of family ancestry in Asia or the specific country/countries of family ancestry in Europe). As much information as was available in the medical record or database was captured in the REA categories, all of which was derived from the patient report. In order to fully represent the breadth of data available, some participants for whom there were more specific ancestral data were stratified into Arab, Ashkenazi Jewish, and Asian country of familial origin. While some groups of Middle Eastern ancestry may self-identify as White, the study team chose to further stratify, as different databases suggest that Arab or Middle Eastern communities may be underrepresented in GWAS (15). This stratification was also performed for those identifying as Hispanic or Latino ethnicity but who may also identify as White. Furthermore, the decision was made to separately analyze individuals of Ashkenazi Jewish ancestry due to the presence of more information on this community from a genomics perspective.

Regarding VUS reclassification, ordering healthcare providers and genetic counselors were notified about reclassifications over time by clinical genetic testing labs either by e-mail and/or portal messages. The VUS reclassification (if any) and the date of reclassification were recorded. Reclassification was determined through a review of the medical record (University of Michigan) or genetics database (KCI) up until December 2023. The research was approved by the University of Michigan Institutional Review Board (IRB) under HUM00223769 and was determined to be non-human subjects research by the Wayne State University/KCI IRB.

### Statistical analysis

Descriptive statistics were presented as counts and percentages for categorical data and as medians (25th to 75th percentile) and mean (SD) for continuous data. Logistic regression was conducted to examine the relationship between REA and VUS reclassification (yes/no). For participants with VUS reclassification, the relationship between REA and time to reclassification was also examined using general linear models. Due to sample size constraints and limited participant numbers in some REA groups, the REA groups analyzed in our models were limited to White, Black or AA, and Asian. A Fisher’s exact test was run to examine the association between participants with more than 1 VUS and REA.

Univariable and multivariable analyses were performed. Participant variables included testing lab, personal history of breast cancer, family history of breast cancer, personal history of other cancer, family history of other cancer, age at the time of genetic testing, year of testing, and number of genes tested. The year of testing and the number of genes tested were not assessed as participant variables in the time to reclassification analysis. Participant variables associated with the outcome at a *p <*0.05 level from the univariable analysis were included in the multivariable analyses. For the time to reclassification analysis, an additional multivariable model was performed to adjust for the baseline year of testing. Due to the exploratory nature of the analysis, multiple comparisons were not performed.

A two-tailed *p*-value of <0.05 was considered statistically significant without adjustment for multiple hypotheses testing. SAS 9.4 statistical software (Cary, North Carolina) was used for all analyses.

## Results

A total of 932 AFAB people were included in the analysis, with 461 from the University of Michigan and 471 from KCI. All participants had at least one VUS, with the large majority having a single VUS (90%) versus two or more concurrent VUS (10%). **Table 1** shows the demographic and clinical characteristics of the study cohort. Of note, the overall mean age was 53 years old at both KCI and the University of Michigan. The overall mean number of genes tested at both institutes was 37.7 (SD = 25.4), and individuals at the KCI had more genes on average per panel (47.8, SD = 29.7) compared to the University of Michigan (27.6, SD = 14.6). Most participants who underwent testing had either a personal history of breast cancer (*n* = 519, 56%) and/or a family history of breast or other cancers (*n* = 485, 52%; *n* = 616, 66%, respectively). Ambry and Invitae were the most commonly used testing companies among both institutions (*n* = 340, 36% and *n* = 506, 54%, respectively).

**Table 1:** Demographics and baseline characteristics by genetic testing site.

Characteristic	Overall (*n* = 932)	Karmanos (*n* = 471)	Michigan (*n* = 461)
Age at the time of testing			
Mean (SD)	53.3 (13.3)	53.5 (13.5)	53.1 (13.1)
Median (IQR)	53.0 (44.0 to 63.0)	53.0 (43.0 to 64.0)	53.5 (44.0 to 63.0)
Range	12.0 to 93.0	12.0 to 93.0	19.0 to 89.0
*N*	931	471	460
Number of genes tested			
Mean (SD)	37.7 (25.4)	47.8 (29.7)	27.7 (14.6)
Median (IQR)	32.0 (19.0 to 48.0)	48.0 (21.0 to 84.0)	25.0 (18.0 to 37.0)
Range	1.0 to 91.0	1.0 to 91.0	1.0 to 84.0
*N*	910	453	457
Genetic testing year			
2013	22 (2)	14 (3)	8 (2)
2014	41 (4)	20 (4)	21 (5)
2015	56 (6)	32 (7)	24 (5)
2016	74 (8)	39 (8)	35 (8)
2017	137 (15)	63 (13)	74 (16)
2018	165 (18)	84 (18)	81 (18)
2019	186 (20)	79 (17)	107 (23)
2020	125 (13)	71 (15)	54 (12)
2021	125 (13)	69 (15)	56 (12)
Lab			
Ambry	340 (36)	213 (45)	127 (28)
GeneDx	1 (0)	0 (0)	1 (0)
Invitae	506 (54)	227 (48)	279 (61)
MMGL	31 (3)	0 (0)	31 (7)
Myriad	39 (4)	16 (3)	23 (5)
Natera	2 (0)	2 (0)	0 (0)
Quest Diagnostics	7 (1)	7 (1)	0 (0)
University of Washington	1 (0)	1 (0)	0 (0)
Missing	5 (1)	5 (1)	0 (0)
Race, ethnicity, and ancestry			
Arab	14 (2)	12 (3)	2 (0)
Ashkenazi Jewish	18 (2)	3 (1)	15 (3)
Asian	48 (5)	4 (1)	44 (10)
Black or African American	144 (15)	121 (26)	23 (5)
Hispanic or Latino	18 (2)	14 (3)	4 (1)
Non-Hispanic or Latino/Pacific Islander	2 (0)	1 (0)	1 (0)
White	634 (68)	270 (57)	364 (79)
Mixed/other	16 (2)	12 (3)	4 (1)
Unknown	38 (4)	34 (7)	4 (1)
Personal history of breast cancer			
No	412 (44)	247 (52)	165 (36)
Yes	519 (56)	224 (48)	295 (64)
Family history of breast cancer			
No	437 (47)	331 (70)	106 (23)
Yes	485 (52)	140 (30)	345 (75)
Unknown	9 (1)	0 (0)	9 (2)
Personal history of other cancer			
	727 (78)	377 (80)	350 (76)
Yes	204 (22)	94 (20)	110 (24)
Family history of other cancer			
No	306 (33)	270 (57)	36 (8)
Yes	616 (66)	201 (43)	415 (90)
Unknown	9 (1)	0 (0)	9 (2)
Number of variants per participant			
1	841 (90)	420 (89)	421 (91)
2	83 (9)	45 (10)	38 (8)
3	7 (1)	5 (1)	2 (0)
4	1 (0)	1 (0)	0 (0)
Number of variants per participant >1			
No	841 (90)	420 (89)	421 (91)
Yes	91 (10)	51 (11)	40 (9)

Categorical measure values are *n* (%).

Regarding REA, the majority of those tested were White (*n* = 672, 72%), with more White participants from the University of Michigan (*n* = 384, 83%) compared to KCI (*n* = 288, 61%). White participants in this group either identified as White race or identified as White race and clarified that they were of single or mixed European ancestry. The second largest group represented was Black or AA people (*n* = 144, 15%), with more Black or AA participants from KCI (*n* = 121, 26%) compared to the University of Michigan (*n* = 23, 5%). The third largest group was Asian people (*n* = 49, 5%), with the majority of these participants undergoing testing at the University of Michigan (*n* = 44, 10%). Family origin data from the University of Michigan cohort among Asian participants were Chinese (*n* = 12, 27%), Indian (*n* = 9, 20%), Japanese (*n* = 6, 14%), Mixed Asian (*n* = 5, 11%), Korean (*n* = 3, 7%), Saudi Arabia (*n* = 1, 2%), Sri Lankan (*n* = 1, 2%), Filipino (*n* = 1, 2%), and Unknown/not reported/not otherwise specified (*n* = 6, 14%). There were five Asian-identifying participants from KCI, with no specific family origin information recorded.

There were a total of 1,032 VUS among the entire study population (with 905 unique variants). Of those VUS that were identified in more than one individual (92 VUS), most were reported twice (70 VUS) and a smaller number were identified three times (17 VUS). The remaining were three of the same VUS reported in four participants, one reported in seven participants, and one reported in nine participants. Compared to White participants with >1 VUS (53/634, 8.4%), Asian and Black participants (7/48, 14.6% and 21/144, 14.6%, respectively) were about twice as likely to have >1 VUS (Fisher’s exact test *p* = 0.038).

**Figure 1:**
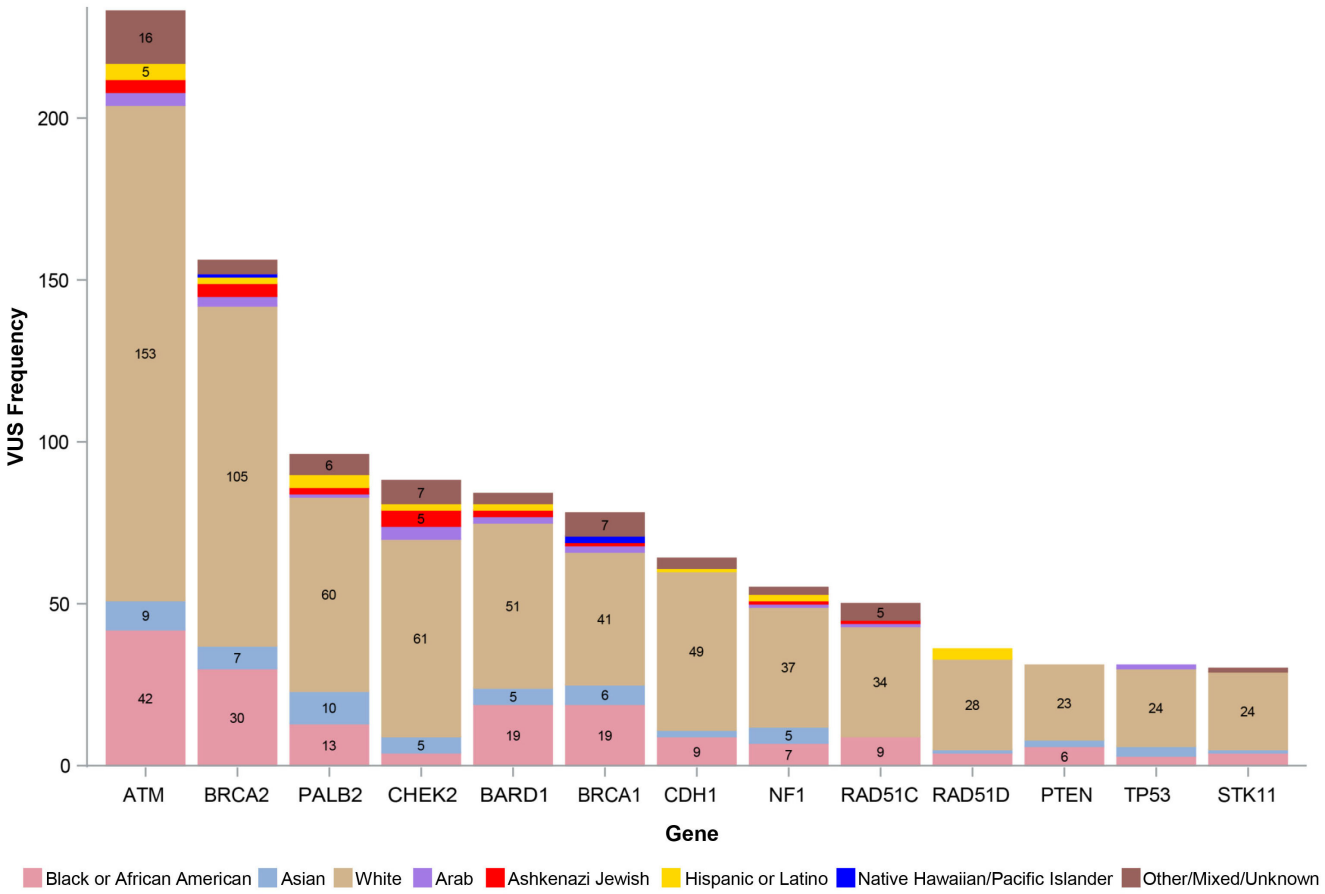
Number of VUS by race, ethnicity, and ancestry. VUS, variants of uncertain significance.

**Figure 2:**
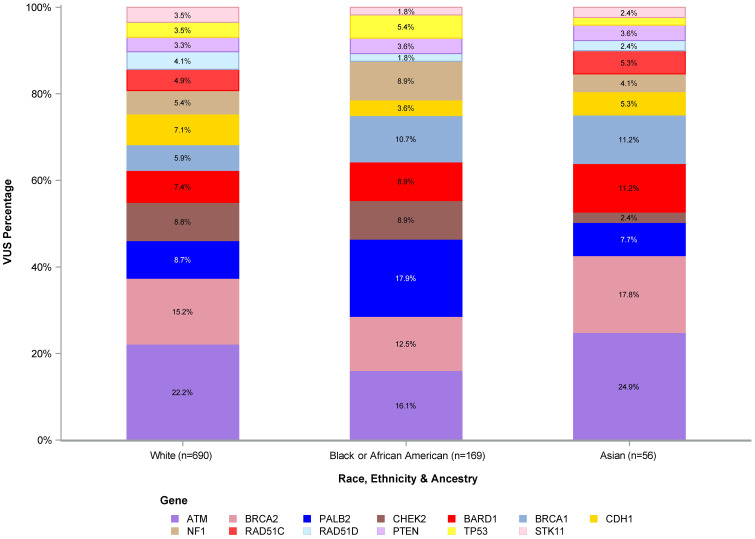
VUS percentage of White, Black or African American, and Asian participants. VUS, variants of uncertain significance.

**Figure 1** shows the number of VUS by REA by gene, with VUS percentage by the most representative REA groups per our study cohort (White, Black or AA, and Asian) by gene depicted in **Figure 2**. Of note, the majority of VUS were observed in larger size genes, specifically *ATM* and *BRCA2* genes (233/1,032, 23% and 156/1,032, 15%, respectively). Regarding the REA distribution of VUS results, White participants represented the majority of *ATM* VUS (153/233, 66%), followed by Black or AA participants (42/233, 18%), and Asian participants (9/233, 4%). White participants also had the majority of *BRCA2* VUS (105/156, 67%), followed by Black or AA participants (30/156, 19%) and Asian participants (7/156, 4%).

Of the total number of VUS, 203 (20%) were reclassified and 831 (80%) were not reclassified as of December 2023. The frequency of VUS reclassifications was similar between institutions (KCI 108/529, 20% and University of Michigan 95/503, 19%). VUS reclassification by REA for the three most represented groups in our cohort was 132/690 (19%) for White participants, 39/169 (23%) for Black or AA participants, and 15/56 (27%) for Asian participants. Regarding Asian participants, there were smaller numbers of VUS reclassification among this group (with ≤3 observed in each gene), although the proportion of VUS reclassification was higher in this group compared to White or Black or AA. REA was not shown to be significantly associated with VUS reclassification within the univariable (*p* = 0.24) or the multivariable analysis (*p* = 0.25). Outside of REA, results from univariable and multivariable analyses are shown in **Table 2**. Results demonstrate that variables such as lab (Ambry), number of genes tested, testing year, family history of breast cancer, and family history of other cancer did show an association with VUS reclassification occurrence in the univariable analysis. However, in the multivariable analysis, only the testing year was statistically significant (OR = 0.81, 95% CI: 0.72 to 0.92, *p* = 0.001).

**Table 2:** Results of logistic regression of VUS reclassification.

Variable	Univariable models		Multivariable model (Univariable *p* < 0.05)	
	OR (95% CI)	*p*-value	OR (95% CI)	*p*-value
Race, ethnicity, and ancestry (Ref: White)	Overall effect	0.244	Overall effect	0.248
Asian	1.55 (0.83 to 2.88)	0.169	1.68 (0.88 to 3.20)	0.119
Black or African American	1.27 (0.85 to 1.90)	0.250	1.19 (0.78 to 1.83)	0.421
Lab (Ref: Invitae)	Overall effect	0.021	Overall effect	0.091
Ambry	1.60 (1.14 to 2.25)	0.007	0.91 (0.59 to 1.39)	0.661
Other	1.06 (0.59 to 1.92)	0.840	0.46 (0.22 to 0.95)	0.036
Age (years) at the time of genetic testing	1.00 (0.99 to 1.01)	0.989		
Number of genes tested	0.99 (0.98 to 0.99)	0.001	1.00 (0.98 to 1.01	0.679
Testing year	0.82 (0.76 to 0.89)	<0.001	0.81 (0.72 to 0.92)	0.001
Personal Hx breast cancer (Ref: no)	1.19 (0.85 to 1.65)	0.307		
Family Hx breast cancer (Ref: no)	1.48 (1.06 to 2.06)	0.021	1.09 (0.73 to 1.62)	0.676
Personal Hx other cancer (Ref: no)	0.88 (0.59 to 1.31)	0.539		
Family Hx other cancer (Ref: no)	1.80 (1.23 to 2.65)	0.003	1.18 (0.71 to 1.96)	0.518

OR, odds ratio; CI, confidence interval; Hx, history; Ref, reference.

**Figure 3:**
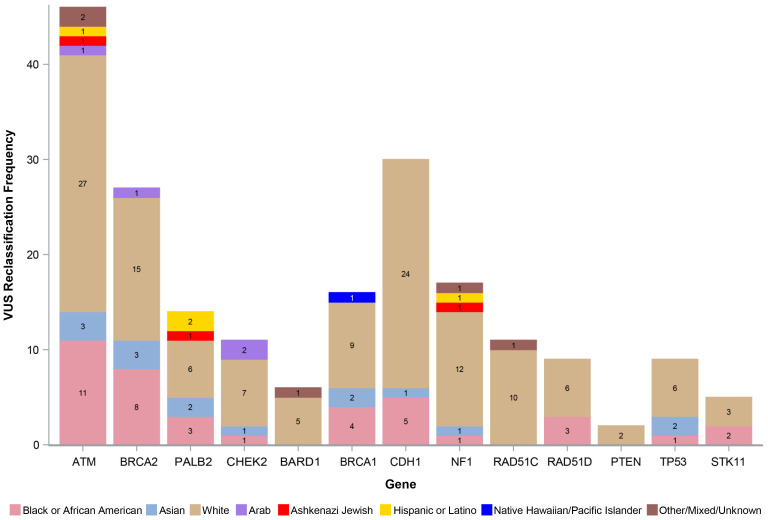
Number of reclassified VUS by race, ethnicity, and ancestry. VUS, variants of uncertain significance.

**Figure 4:**
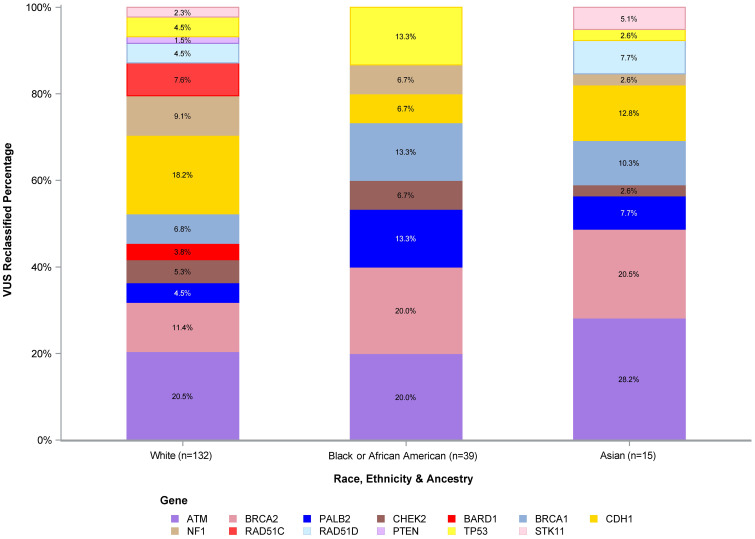
VUS reclassified percentage of White, Black or African American, and Asian participants. VUS, variants of uncertain significance.

The number of VUS reclassified by REA by gene is shown in **Figure 3**, with the percentage of reclassified VUS by the three most represented REA groups in our study cohort (White, Black or AA, and Asian) depicted in **Figure 4**. The genes with the highest number of reclassifications were *ATM* (46/233, 20%) and *CDH1* (30/64, 47%). The reclassification of *ATM* VUS was as follows: White 27/153 (18%), Black or AA 11/42 (26%), and Asian 3/9 (33%). The reclassification of *CDH1* VUS was as follows: White 24/49 (49%), Black or AA 5/9 (56%), and Asian 1/2 (50%).

The mean time to VUS reclassification across all groups was 2.8 years. There were similar reclassification durations between the two institutions, with KCI mean time being 2.9 years (SD = 1.7) and the University of Michigan mean time being 2.8 years (SD = 2.0). The results from univariable and multivariable analyses assessing time to VUS reclassification are shown in **Table 3**. There was a significant association noted between time to VUS reclassification and testing lab, with an overall effect of *p <*0.001. Ambry and all the other labs had a longer time to VUS reclassification compared to Invitae (estimate = 0.73 years, 95% CI: 0.20 to 1.25, *p* = 0.007 for Ambry; estimate = 2.34 years, 95% CI: 1.43 to 3.24, *p* < 0.001 for all the other labs). However, with adjustment for the baseline testing year, this overall effect was no longer significant (*p* = 0.05).

**Table 3:** General linear model results of time to reclassification of VUS.

Variable	Univariable models		Multivariable model (univariable *p* < 0.05)		Multivariable model (adjusted for baseline testing year)	
	Estimate (95% CI)	*p*-value	Estimate (95% CI)	*p*-value	Estimate (95% CI)	*p*-value
Race, ethnicity, and ancestry (Ref: White)	Overall effect	0.059	Overall effect	0.158	Overall effect	0.216
Asian	0.71 (−0.25 to 1.67)	0.148	0.70 (−0.19 to 1.60)	0.124	0.68 (−0.11 to 1.46)	0.093
Black or African American	0.69 (0.04 to 1.34)	0.037	0.44 (−0.18 to 1.07)	0.166	0.21 (−0.34 to 0.77)	0.452
Lab (Ref: Invitae)	Overall effect	<0.001	Overall effect	<0.001	Overall effect	0.050
Ambry	0.88 (0.37 to 1.38)	0.001	0.73 (0.20 to 1.25)	0.007	−0.40 (−0.95 to 0.16)	0.158
Other	2.49 (1.59 to 3.38)	<0.001	2.34 (1.43 to 3.24)	<0.001	0.49 (−0.46 to 1.42)	0.310
Age (years) at the time of genetic testing	−0.02 (−0.04 to 0.01)	0.140				
Personal Hx breast cancer (Ref: no)	0.46 (−0.08 to 0.99)	0.092				
Family Hx breast cancer (Ref: no)	0.71 (0.17 to 1.24)	0.010	0.49 (−0.02 to 0.99)	0.058	0.16 (−0.29 to 0.61)	0.489
Personal Hx other cancer (Ref: no)	−0.27 (−0.92 to 0.38)	0.408				
Family Hx other cancer (Ref: no)	0.42 (−0.22 to 1.07)	0.196				
Baseline testing year					−0.54 (−0.69 to −0.40)	<0.001

Estimates, change in time to reclassification per 1 unit change in the predictor.

CI, confidence interval; Hx, history; Ref, reference; 95% CI, 95% confidence interval.

The mean time to reclassification from the univariable analysis was 2.6 years (95% CI: 2.3 to 2.9), 3.3 years (95% CI: 2.7 to 3.9), and 3.3 years (95% CI: 2.4 to 4.2) for White, Black or AA, and Asian participants, respectively. Of note, the univariable analysis demonstrated that Black or AA had a longer time to reclassification by 0.7 years compared to White participants (*p* = 0.037), though there was no significant overall effect (*p* = 0.059). After adjusting for potential confounders (testing laboratory and family history of breast cancer), the overall association between REA and time to reclassification remained non-significant (overall effect *p* = 0.16). The least square (LS) means were as follows: White LS mean = 3.1, 95% CI: 2.7 to 3.5; Black or AA LS mean = 3.5, 95% CI: 2.9 to 4.1; and Asian LS mean = 3.8, 95% CI: 2.9 to 4.7.

**Figure 5:**
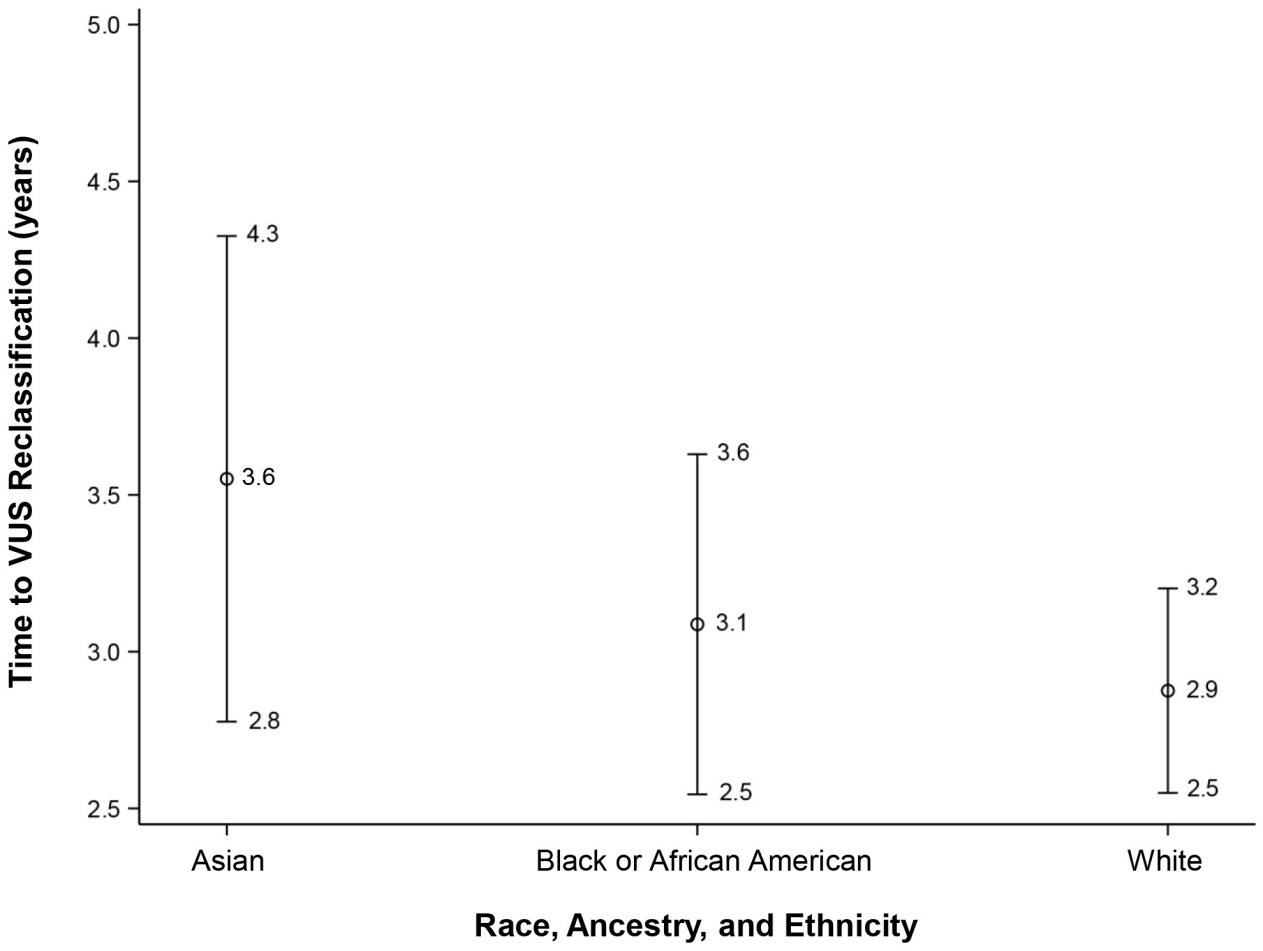
Time to VUS reclassification for White, Black or African American, and Asian participants. VUS, variants of uncertain significance.

After adjusting the multivariable model for the baseline testing year, the association with REA remained non-significant for time to reclassification (overall effect *p* = 0.22). The LS means were as follows: White LS mean = 2.9, 95% CI: 2.5 to 3.2; Black or AA LS mean = 3.1, 95% CI: 2.5 to 3.6; and Asian LS mean= 3.5, 95% CI: 2.8 to 4.3, as represented in **Figure 5**. Of note, there was no significant association observed with time to reclassification for VUS downgrading to benign/likely benign (mean = 2.9, 95% CI: 1.7 to 2.0) versus those VUS upgrading to pathogenic/likely pathogenic (mean = 2.4, 95% CI: 1.4 to 3.4) (*p* = 0.36).

Regarding the type of reclassification, the majority of VUS were reclassified to benign (*n* = 83, 41%) and likely benign (*n* = 104, 51%), for a total of 92% of all reclassified VUS being downgraded. Most overall downgrades occurred in *ATM* (44/187, 23.5%), *CDH1* (30/187, 16.0%), and *BRCA2* (26/187, 13.9%). Reclassifications to benign included *ATM* (*n* = 19), *BRCA1* (*n* = 6), *BRCA2* (*n* = 13), *CDH1* (*n* = 10), *CHEK2* (*n* = 2), *NF1* (*n* = 12), *PALB2* (*n* = 6), *RAD51C* (*n* = 3), *RAD51D* (*n* = 4), *STK11* (*n* = 4), and *TP53* (*n* = 4). Reclassifications to likely benign included *ATM* (*n* = 25), *BARD1* (*n* = 5), *BRCA1* (*n* = 5), *BRCA2* (*n* = 13), *CDH1* (*n* = 20), *CHEK2* (*n* = 5), *NF1* (*n* = 5), *PALB2* (*n* = 8), *PTEN* (*n* = 2), *RAD51C* (*n* = 8), *RAD51D* (*n* = 3), *STK11* (*n* = 1), and *TP53* (*n* = 4). A total of 16 participants had VUS that were reclassified to actionable pathogenic/likely pathogenic variants (8% of the total VUS reclassified). Overall, most upgrades occurred in *BRCA1* and *CHEK2* although these were present in very small numbers (*n* = 5 and *n* = 4, respectively). White participants represented the majority of those who had VUS that were upgraded (*n* = 11). Pathogenic upgrades among White participants included *BARD1* (*n* = 1), *BRCA1* (*n* = 3), *BRCA2* (*n* = 1), and *CHEK2* (*n* = 1). Likely pathogenic upgrades among White participants included *ATM* (*n* = 1), *CHEK2* (*n* = 2), and *RAD51D* (*n* = 2). Of the total Black or AA participants, three had VUS that were upgraded (one likely pathogenic variant in *BRCA1*, one pathogenic variant in *BRCA1*, and one pathogenic variant in *TP53*). There was one Asian participant with an *ATM* VUS that was upgraded to likely pathogenic and one Arab participant with a *CHEK2* VUS that was upgraded to pathogenic.

Of note, one additional White participant with a *BRCA1* VUS was clinically reclassified as pathogenic despite being persistently categorized as a VUS by the participant’s testing laboratory due to the participant’s mother having the same VUS reclassification to pathogenic by another laboratory (in the context of a strong family history of breast cancer).

## Discussion

Although it is generally accepted that most VUS are reclassified to benign/likely benign, this principle has not been widely studied among communities of non-European ancestry. There are scarce data demonstrating if and how these VUS are reclassified and the length of time to reclassification for non-European populations.

Our study demonstrated several key findings. White participants represented the largest REA group in our cohort, which could explain why this group represented the large majority of VUS carriers. *ATM* and *BRCA2* were the most represented VUS, which may be a function of their large gene size. While normalization of the total length of gene regions (such as coding regions and adjacent intron sequences) could be considered, this was outside the scope of this study. Regarding reclassification, there was a greater proportion of VUS reclassification among Black or AA and Asian participants compared to White participants. Asian participants had the highest proportion of VUS reclassified although their VUS were present in overall smaller numbers. There was no statistically significant difference between VUS reclassification and REA. Black or AA and Asian participants had a slightly longer time to reclassification, but this was also not statistically significant. The majority of VUS in this cohort were downgraded to benign/likely benign, which is consistent with prior literature (27–30, 34). Only a small percentage (8%) were reclassified to pathogenic/likely pathogenic. These numbers were too small to perform any statistical analysis, although it was observed that most reclassification to pathogenic/likely pathogenic occurred among White participants. Again, this could be attributed to this group being represented in larger numbers but this is still unclear.

The reason for *BRCA* genes being more frequently reclassified as pathogenic/likely pathogenic is unclear, although the numbers are still relatively small. This could highlight the overall increased population prevalence and increased breadth of research on *BRCA1/2* variants. Regarding the *TP53* variant upgrade, our understanding and knowledge of *TP53* variants have evolved over time. Not all variants of *TP53* are well described. The interpretation of germline *TP53* variants integrates epidemiological and phenotypical bioinformatics prediction and functional data. The penetrance of germline disease-causing *TP53* variants is variable, depending partially on the type of variant (dominant-negative variants being associated with a higher cancer risk). Even though this pathogenic *TP53* reclassification only involved one participant, the impact of *TP53* diagnosis is tremendous regarding cancer risk and screening. Such a diagnosis has significant implications given the high overall cancer risk with this particular gene. Interestingly, this reclassification occurred in a Black or AA patient, which continues to underscore the need for more testing and research in this community.

As the volume of literature on this topic is limited, these results have significant implications. Our results support the high likelihood of reclassification to benign/likely benign among all individuals undergoing genetic testing regardless of REA and therefore have the potential to provide more reassurance to patients during the counseling process regardless of a higher possibility of VUS occurrence due to REA. These findings also highlight that VUS may still carry a small possibility of upgrading to pathogenic/likely pathogenic, in which clinical care may change dramatically. Patients must still undergo appropriate counseling and must be followed closely over time for any changes in VUS that would be clinically actionable. Additionally, as with one participant in this cohort, a VUS may be clinically upgraded based on supplemental data of close relatives in the setting of a strong family history of breast cancer.

There were several strengths to this study. This study represents one of the first assessing VUS reclassification among a diverse cohort and (to our knowledge) the first study assessing the time to and type of VUS reclassification exclusively among AFAB people at risk for breast cancer. The study addresses a very important and largely unanswered research question regarding genetic testing among individuals from diverse REA groups. Furthermore, the University of Michigan and KCI represent two large institutions with strong genetics programs that provide genetic testing to regionally diverse cohorts in two metropolitan areas of Ann Arbor and Detroit, Michigan. Although the majority of people in this cohort who underwent genetic testing were of European ancestry, the two institutions included in this study serve diverse groups that allowed for some comparison.

There were also limitations. The small numbers of certain REA groups (such as Arab, Ashkenazi Jewish, Hispanic or Latino, and non-Hispanic or Latino/Pacific Islander) limited our analysis of those communities. Despite the relative diversity of the study, there were low numbers of Arab participants observed. While it is unclear if the number of VUS among this group was a proxy for overall genetic testing, it is similarly unclear if the results from this study are generalizable, particularly in light of the large representation of Arab communities in the state of Michigan (second highest populous state after California) (35). It is also possible that some Arab participants may have been identified under White race (as per NIH classification) and were not stratified to Arab due to lack of reported ancestry data.

This calls to attention the need for more systematic documentation of ancestry in genetic encounters and throughout the medical system to gain more clarity in understanding individual genetics. Even with such efforts, REA is often self-reported—as was in this study—which is subject to participant bias or uncertainty. We also recognize that knowledge of direct ancestry is a privilege not available to all groups. This is the case, for instance, with some Black communities in the United States who may identify as AA but may not know their exact country or region of ancestry in Africa due to ancient migrations as well as generations of forced displacement from the transatlantic slave trade. While we recognize that ancestry is a preferable population descriptor over race and ethnicity (36), this information may not always be readily available for all communities. This study, therefore, aimed to comprehensively include all available REA population descriptors for a more robust understanding and interpretation of this diverse cohort.

The general process of VUS reclassification should also be more closely examined. All clinical laboratories utilize guidelines published by the American College of Medical Genetics (ACMG) and the Association for Molecular Pathology (AMP) for initial variant classification (37). Variants are classified as benign/likely benign when evidence demonstrates a >90% certainty that they are not associated with disease, and similarly, variants are classified as pathogenic/likely pathogenic if they are determined to increase the risk of disease with >90% certainty. VUS classification occurs when there are not enough data to classify as benign/likely benign or pathogenic/likely pathogenic (37). However, regarding VUS reclassification, there is currently no standard method across laboratories. There are some genetic testing laboratories that use an “active” approach to reclassification. This entails computational reviews of all variants systematically to ensure continuous reassessment of variants. Another approach involves more “passive” reclassification, which relies on a variety of data sources. This could include providers giving information to testing companies on clinical phenotype–genotype correlations, literature review as new data becomes available, bioinformatics, interval genome reanalysis, and family studies (27, 38). There is a need for standardization of VUS reclassification across laboratories to decrease the volume of conflicting interpretations and confusion regarding clinical management. These discrepancies can be appreciated in databases such as ClinVar, for which there can be varying interpretations of a single variant. An example of this discrepancy was highlighted in our study participant whose VUS was treated as pathogenic due to her mother’s testing company interpreting the variant as pathogenic (despite the participant’s lab that classified the same variant as a VUS).

There are several considerations regarding results interpretation and clinical management. There are rare instances in which VUS may be clinically treated as pathogenic/likely pathogenic based on the molecular characteristics of the change (e.g., highly conserved amino acid changing to a highly dissimilar amino acid) that raise suspicion of being potentially structurally disruptive. These considerations are rare, and when they occur, it is usually in the context of a strong family history of cancer and other data regarding the VUS. Furthermore, some participants in this cohort may have had concurrent actionable results in another breast cancer susceptibility gene or may have had a calculated increased breast cancer risk based on family history that would not have necessarily changed their clinical management whether or not their VUS were reclassified. However, this information was not included in order to focus on the rates and types of reclassification among VUS alone such that these VUS were not dependent on findings from other genes, but rather viewed as independent events and data.

It is important to recognize the limitations of the data that are used for reclassification. While the results from this study suggest that VUS among diverse groups are mostly reclassified to benign/likely benign (which is largely consistent with the data from predominantly European populations), these reclassification data are still largely based on European study populations. There could be some inherent bias for which the variants of diverse communities are not being fully explored and assessed in the context of those for whom they share common ancestry. This underscores the importance of continuing to work toward more inclusive genetic databases.

Furthermore, VUS reclassification or lack thereof could have implications on patient care. While some prospective studies suggest fewer psychosocial problems related to a VUS diagnosis (possibly related to the nature of post-test counseling) (39) and similar scores in the Multidimensional Impact of Cancer Risk Assessment (MICRA) compared to those with negative results (40), other studies showed increased MICRA scores (41) and increased patient distress with VUS results (42). These data illuminate the potential psychological impact on patients which could be an important consideration in counseling.

This research evaluating VUS reclassification among a diverse cohort at risk for breast cancer showed no significant differences in VUS reclassification occurrence and time to reclassification by REA. There were no significant differences in time to and type of VUS reclassification by REA. Most VUS were reclassified to benign/likely benign, with a small percentage upgrading to pathogenic/likely pathogenic. This study could serve to enhance and guide communication to diverse communities undergoing genetic testing, allowing for more informed and equitable care. Our research also reinforces the continuing need for greater inclusion of diverse communities in genetic studies.

## Data Availability

The data analyzed in this study is subject to the following licenses/restrictions: One database was preexisting and other was collection of retrospective data. Requests to access these datasets should be directed to vershap@med.umich.edu.
