# Supplementary material for: Reclassification of variants of uncertain significance by race, ethnicity, and ancestry for patients at risk for breast cancer
Source: Front Oncol. 2025 Feb 18;15:1455509. doi: 10.3389/fonc.2025.1455509 (PMC11876048; doi:10.3389/fonc.2025.1455509)
Supplement: Supplementary file 1 [file Table1.docx]

**Supplemental Table – Number of VUS by Gene within Race, Ethnicity & Ancestry Groups**

| **Gene** | **White** | **Black  or AA** | **Asian** | **Hispanic or Latino** | **Ashkenazi  Jewish** | **Arab** | **NH/PI** | **Mixed, Other or Uknown** | **Total** |
| --- | --- | --- | --- | --- | --- | --- | --- | --- | --- |
| **ATM** | 153 | 42 | 9 | 5 | 4 | 4 | 0 | 16 | 233 |
| **BRCA2** | 105 | 30 | 7 | 2 | 4 | 3 | 1 | 4 | 156 |
| **PALB2** | 60 | 13 | 10 | 4 | 2 | 1 | 0 | 6 | 96 |
| **CHEK2** | 61 | 4 | 5 | 2 | 5 | 4 | 0 | 7 | 88 |
| **BARD1** | 51 | 19 | 5 | 2 | 2 | 2 | 0 | 3 | 84 |
| **BRCA1** | 41 | 19 | 6 | 0 | 1 | 2 | 2 | 7 | 78 |
| **CDH1** | 49 | 9 | 2 | 1 | 0 | 0 | 0 | 3 | 64 |
| **NF1** | 37 | 7 | 5 | 2 | 1 | 1 | 0 | 2 | 55 |
| **RAD51C** | 34 | 9 | 0 | 0 | 1 | 1 | 0 | 5 | 50 |
| **RAD51D** | 28 | 4 | 1 | 3 | 0 | 0 | 0 | 0 | 36 |
| **PTEN** | 23 | 6 | 2 | 0 | 0 | 0 | 0 | 0 | 31 |
| **TP53** | 24 | 3 | 3 | 0 | 0 | 1 | 0 | 0 | 31 |
| **STK11** | 24 | 4 | 1 | 0 | 0 | 0 | 0 | 1 | 30 |
| **Total** | 690 | 169 | 56 | 21 | 20 | 19 | 3 | 54 | 1032 |

Abbreviations: VUS, Variants of Uncertain Significance; AA, African American; NH/PI, Native Hawaiian and Pacific Islander
